# The effect of face interpretation training on social anxiety symptoms

**DOI:** 10.3389/fpsyg.2026.1735938

**Published:** 2026-05-19

**Authors:** Grace L. Wheeler, Shari A. Steinman

**Affiliations:** 1 Department of Psychology, West Virginia University, Morgantown, WV, United States; 2 Department of Psychological Science, University of Vermont, Burlington, VT, United States

**Keywords:** cognitive bias modification, emotion recognition, face interpretation bias modification, FIBM, social anxiety

## Abstract

**Introduction:**

According to cognitive models, individuals with social anxiety place excessive importance on gaging others’ reactions to their social performance. This may lead to misinterpretation of facial expression. Facial interpretation bias modification (FIBM) was developed to shift interpretations of faces.

**Methods:**

The current study uses FIBM to test whether face interpretation is causally related to social anxiety symptoms in an unselected sample. Participants (*N* = 139) were randomized to 1 of 3 conditions (positive, negative, accurate) in a single-session, online study. Pre- and post-intervention measures of face interpretation and social anxiety were completed.

**Results:**

Individuals in the positive condition interpreted faces more positively at post-test compared to baseline, and individuals in the negative condition interpreted faces more negatively at post-test compared to baseline. Contrary to hypotheses, FIBM did not affect social anxiety symptoms.

**Conclusion:**

It is possible the interpretation of faces may not be related to social anxiety.

## Introduction

The defining feature of social anxiety disorder (SAD) is an intense fear or anxiety regarding social situations in which the individuals may be judged by others (American Psychiatric Association, 2013). The cognitive model of social anxiety posits people develop social anxiety if they attach excessive importance to evaluation from others (Rapee and Heimberg, 1997). Individuals with SAD constantly scan the audience for potential negative reactions, which leads to increased anxiety and misidentification of ambiguous cues from the audience. Neutral or ambiguous expressions are often interpreted as negative or threatening (Yoon and Zinbarg, 2008). This negative interpretation then furthers anxiety, acting as a negative feedback loop.

Cognitive bias modification (CBM) was developed to test the theory that cognitive processes (e.g., negative interpretation, attention to threat) cause anxiety (Beck et al., 1985). In CBM, cognitive biases are directly manipulated through repeated exercises aimed to counteract negative predispositions and subsequent effects on anxiety are measured. Meta-analyses and reviews suggest CBM for interpretations (MacLeod and Mathews, 2012; Krebs et al., 2018) and attention (Fodor et al., 2020; Hakamata et al., 2010; MacLeod and Mathews) leads to a reduction in social anxiety and anxiety symptoms. However, some have found effects to be small (Liu et al., 2017; Fodor et al., 2020) or inconsistent (Fodor et al., 2020; Heeren et al., 2015). Studies suggest that multiple training sessions of CBM may lead to larger treatment effects (Hallion and Ruscio, 2011; Krebs et al., 2018; Jones and Sharpe, 2017; Hakamata et al., 2010; Beard et al., 2012). However, Fodor et al. (2020) found that number of sessions was not associated with treatment outcomes, but that lab delivery of CBM was associated with better outcomes (opposed to delivery online, in a hospital, or a combination). CBM effects are not moderated by age or gender (Hakamata et al., 2010). More recently, researchers have developed a CBM treatment that uses facial interpretation training called Facial Interpretation Bias Modification (FIBM; Penton-Voak et al., 2012). In this protocol, a CBM task is designed to shift the interpretations of facial expressions (Kuin et al., 2020; Penton-Voak et al., 2012; Penton-Voak et al., 2020; Peters et al., 2017; Van Meter et al., 2021).

The current study aimed to replicate and extend research on FIBM tasks. In FIBM, participants complete baseline, training, and post-test. During baseline, participants are presented one of 15 faces which range from two different emotions (e.g., happy to angry) on a 15-face scale and categorize the emotion. Using this baseline data, researchers establish a balance point (i.e., the point within the 15-face range the participant identified the face as happy instead of angry), or FIBM score, for each participant. During training, participants are presented with the faces again, but given feedback (“correct” or “incorrect”) on their categorization of the emotion. Feedback is based on shifting the balance point by two increment faces on the 15-face continuum (e.g., if they had a FIBM score of five in the baseline trial, they received feedback to interpret the first seven faces in the morph as positive), inducing an interpretation bias. During post-test, participants are presented with the same faces and categorize them; they are not given feedback. Post-test reveals the effect of the training on facial interpretations, and whether a bias is induced.

There is a range of protocol durations (one day to one week) within FIBM studies (Kuin et al., 2020; Penton-Voak et al., 2013). Significant change in face interpretation has been demonstrated in both shorter-term and longer-term durations. The current study uses a single-session format to decrease potential confounding variables between sessions and decrease participant attrition. Across studies, there is evidence that FIBM task effects are maintained at 2-week follow up (Penton-Voak et al., 2012; Rawdon et al., 2018), 6-week follow up (Penton-Voak et al., 2020; Kuin et al., 2020), and 8-week follow up (Van Meter et al., 2021). Of the three studies that observed significant FIBM effects on mood, only Rawdon et al. (2018) and Van Meter et al. (2021) assessed mood impacts at follow-up; they found that effects were maintained at 2-weeks and 8-weeks following intervention, respectively.

FIBM has been used in healthy samples as well as clinical samples (Kuin et al., 2020; Penton-Voak et al., 2012; Penton-Voak et al., 2020; Peters et al., 2017). Studies have demonstrated a significant change in face interpretations, in which participants experienced a positive shift in interpretation bias as a result of training. However, most of these studies have not shown significant effects on measures of psychopathology, such as state and trait anxiety, depression, stress, anger, or behavioral outcomes on prosocial behavior and aggressive behavior. This may be because the interpretation of ambiguous faces is not theorized to be a key causal mechanism of these forms of psychopathology. In the current study, we expected to see effects of FIBM on social anxiety because facial interpretation for audience evaluation is one of the core tenets in the social anxiety cognitive model (Rapee and Heimberg, 1997).

Notably, three studies did find an effect of FIBM on psychopathology. Penton-Voak et al. (2013) demonstrated an FIBM balance point change in a one-session intervention on healthy adults and high-risk youth using a happiness to anger continuum. They also found a decrease in self-reported and observer-reported anger and aggression after a four-to-five-day FIBM intervention in high-risk youth. Van Meter et al. (2021) used a three-session FIBM protocol and trained young adult participants (ages 16–25) on a happiness to sadness continuum. Results observed a shift in FIBM balance point and a decrease in self-reported depression at study conclusion and 8-week follow up. Rawdon et al. (2018) was the only study to assess FIBM on social anxiety and did so in an adolescent sample (ages 15–18). This study trained individuals on a happiness to disgust facial expression continuum. They found an impact in face interpretations and a decrease in depressive symptoms at two-week follow up but found no change in social anxiety symptoms. It’s unclear why FIBM affected depression in this study, given the intervention’s focus on social anxiety, and null results on depression and other mood measures in previous studies. This result suggests FIBM can affect psychopathology symptoms, though effects on psychopathology are less common than effects on face interpretations.

The current one-session proof of principle study was the first to assess FIBM effects on social anxiety symptoms in adults, and the first to evaluate negative and accurate FIBM conditions in any participant sample. The negative interpretation bias induction tested the theorized causal relationship between interpretation of audience expressions and symptoms of SAD (Rapee and Heimberg, 1997). An accurate condition was included to test if improved accuracy (instead of positive bias) might reduce social anxiety symptoms.

Methodological issues in previous research may have led to nonsignificant results on psychopathology symptoms. Several changes were made in the current study to strengthen FIBM impact on social anxiety symptoms. First, the current FIBM task used more relevant stimuli (e.g., happiness to anger, as opposed to happiness to disgust, as done in Rawdon et al., 2018) and theoretically relevant measures (e.g., social anxiety measures, as opposed to anger or depression, as done in Kuin et al., 2020 and Penton-Voak et al., 2012). Previous research has demonstrated a sensitivity for anger recognition in individuals with high social anxiety (Cui et al., 2021). Second, before starting the task, participants were instructed to imagine they were giving a presentation, and the following faces were faces in the audience, to simulate a real-life scenario in assessing audience reaction and be more closely tied to the cognitive model of social anxiety (Rapee and Heimberg, 1997). Previous articles did not include specific instructions given to participants before starting FIBM tasks. Third, current FIBM task increased the balance-point shift to include the entire middle range of five faces (as opposed to creating an individualized balance point and only shifting two faces past the balance point as done in previous FIBM studies; Peters et al., 2017). This means that participants were trained to interpret the middle five faces in the continuum per their condition assignment (e.g., negative, positive, or accurate). This change may lead to more dramatic shift in interpretation biases and bolster potential impacts seen on social anxiety. Fourth, faces remained on screen until the participant answered the categorization question to evaluate interpretation of facial expressions instead of evaluating potential memory bias if the faces were no longer visible (as done in previous FIBM studies; Peters et al., 2017).

It was hypothesized that following FIBM, participants in the positive condition would have an increase in FIBM scores, indicating they interpreted more faces as happy, while participants in the negative condition would have a decrease in FIBM scores, indicating they interpreted fewer faces as happy. Furthermore, at post-intervention, it was hypothesized social anxiety scores would be lowest in the positive condition, then accurate condition, and highest in the negative condition. In exploratory analyses, age was tested as a covariate and Fear of Positive Evaluation Scale score and baseline social anxiety were evaluated as moderators for all outcomes.

## Methods

### Participants

**Figure 1:**
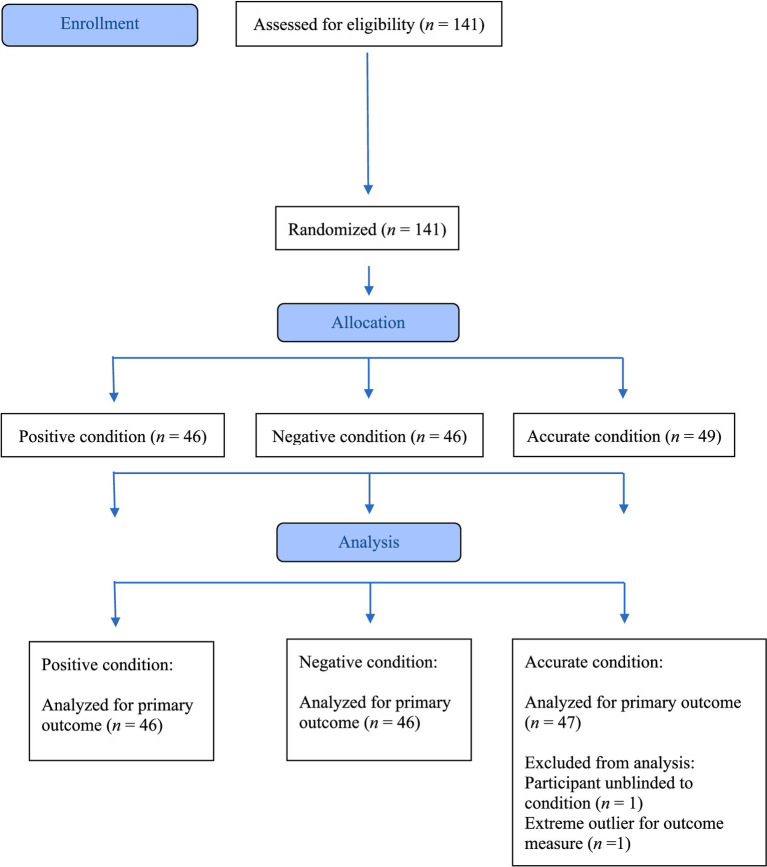
CONSORT 2025 flow diagram. Flow diagram of the progress through the phases of a randomized trial of three groups (that is, enrollment, condition assignment, and data analysis).

The sample included 141 adults recruited through a psychology department participant pool and a university survey listserv (Figure 1). To be eligible for the study, potential participants needed to be 18 years of age or older and comfortable reading and writing in English. Eligibility was assessed at onset of study appointment. All participants who were eligible and consented to study procedure completed the task and measures. Sample size was determined by a power analysis for a repeated measures Analysis of Variance (ANOVA) with a within-between interaction, with the number of groups (3) entered as a between-group factor and the number of timepoints (2) entered as a within-group factor using G*power analysis (Faul et al., 2007). Given null effects in previous studies, a power analysis to detect a small effect size (*f* = 0.15) with a power of 0.85 (alpha = 0.05) was conducted, leading to an optimal *N* of at least 126. We excluded two participants from all analyses: one participant was excluded due to researcher error (i.e., participant was told their condition assignment prior to the intervention), and another was an extreme outlier for their FIBM scores (FIBM baseline and post-test scores were more than three times the interquartile range from the first quartile). Therefore, data from 139 individuals were analyzed among the negative (*n* = 46), positive (*n* = 46), and accurate conditions (*n* = 47). See Table 1 for demographic information.

**Table 1:** Demographic composition of study conditions.

Demographic variable	Positive		Negative		Accurate	
	M	SD	M	SD	M	SD
Age	22.87	5.00	24.13	8.50	20.62	3.34
	Percentage	N	Percentage	N	Percentage	N
Gender						
Cisgender female	63.04	29	63.04	29	68.09	32
Cisgender male	28.26	13	30.43	14	27.66	13
Non-binary	6.52	3	6.52	3	2.13	1
Transgender male	2.17	1	0	0	0	0
Other	0	0	0	0	2.13	1
Race						
White	69.57	32	7.61	35	70.21	33
Asian/Pacific Islander	13.04	6	13.04	6	4.26	2
Black/African American	2.17	1	2.17	1	6.38	3
Multi-racial	13.04	5	4.34	2	14.89	7
Other	4.34	2	4.34	2	4.26	2
Ethnicity						
Non-Hispanic	93.48	43	86.96	40	95.74	45
Hispanic	6.52	3	13.04	6	4.26	2

### Measures

#### Baseline measures

##### Demographics

We collected age, gender, race, and ethnicity for all participants.

Fear of Positive Evaluation Scale (FPES) was used to assess baseline fear of positive evaluation (Weeks et al., 2008). The FPES is a 10-item self-report assessment. Items refer to social situations in which the individual would be subjected to positive evaluation (Weeks et al., 2008). The FPES has demonstrated good psychometrics (Weeks et al., 2008). In the current study, the FPES had strong internal consistency (*α* = 0.82).

Social Interaction Anxiety Scale (SIAS) was used to assess baseline social anxiety and FIBM effects on social anxiety (Mattick and Clarke, 1998). Item 14 was adjusted to be inclusive of non-heterosexual orientations. The SIAS is a 20-item, self-report instrument and presents statements and the participant rates how characteristic the statement is to them. The SIAS has demonstrated good psychometrics (Carter et al., 2014; Mattick and Clarke, 1998). The SIAS has also shown to be a sensitive measure for one session studies evaluating effects on anxiety (Possis et al., 2013). In the current study, the SIAS demonstrated strong internal consistency (baseline *α* = 0.92; post-intervention assessment *α* = 0.95).

##### Task

FIBM was used to train participants in the negative, positive, and accurate conditions for expression categorization. The FIBM task in the current study used 15 composite faces morphing from happy to angry from images in the Karolinska Directed Emotional Faces (KDEF; Lundqvist et al., 1998) and used a similar procedure to Penton-Voak et al. (2012). FIBM was presented via Qualtrics in one session with three phases: baseline, training, and post-test.

Prior to the baseline phase, participants received the following instructions, *“Please imagine you are giving a speech in front of a crowd. The faces you will see are faces from the audience. You will be asked to choose which label best describes the face presented from several options.”* For training and post-test, participants were asked to remember the previous instructions. For the negative and positive conditions’ baseline, participants were presented with a face in the center of the screen and instructed to categorize the expression of the presented face in a two-option force choice procedure (e.g., “approving,” “criticizing”) below the face in each trial. Participants in the accurate condition were given a three-option forced choice procedure (e.g., “approving,” “ambiguous,” “criticizing”). Each face was presented three times (45 trials) and the face remained on the screen for 60 s or until the participant selected a response. If a response was not selected within 60 s, the trial automatically proceeded to the next face. During baseline, participants did not receive feedback.

During training, participants were presented with the same 15 faces and forced choice response. Each face was presented twice (30 trials). Participants were then provided feedback on each trial (e.g., “Correct! That face was approving”, “Incorrect! That face was criticizing”) based on their condition; feedback remained for 1 s. Participants in the positive condition were trained to interpret the first five faces and the middle five faces as “approving” rather than criticizing. Participants in the negative condition were trained to interpret the first five faces and the middle five faces as “criticizing” rather than approving. Participants in the accurate condition were trained to interpret the faces based on the categorization that the first five faces were “approving”, the middle five faces were “ambiguous”, and the last five faces were “criticizing.” During post-test, each face was presented three times (45 trials), but participants were not provided feedback.

##### FIBM scoring

FIBM is scored by the number of faces categorized as happy divided by 45 (total number of trials in phase), then multiplied by 15 (number of unique faces in trial), and finally rounded to the nearest whole integer (Penton-Voak et al., 2012; Peters et al., 2017). The accuracy condition was analyzed separately with a paired samples *t*-test given its use of three (vs. two) response choices. The “approving” choice was coded as correct (“1”) for the first five faces, the “ambiguous” choice was coded as correct (“1”) for the middle five faces, and the “criticizing” choice was coded as correct (“1”) for the last five faces. The correct choices were summed, divided by 45 (total number of trials in phase), multiplied by 15 (number of unique faces in trial), and rounded to the nearest whole integer for a final accuracy score.

##### Post-intervention measures

Liebowitz Social Anxiety Scale – Self Report (LSAS-SR) was used to assess post-intervention effects of FIBM on social anxiety (Baker et al., 2002). It is possible that individuals completing the post-test SIAS would recall their responses to the measure at baseline and thus would not change their responses at post-test. Therefore, the LSAS-SR was included as an additional post-test anxiety measure that was novel to participants at post-test. The LSAS-SR is a 24-item, self-report instrument measuring social anxiety in respondents (Baker et al., 2002). Instructions were modified to focus on anticipated reactions in future scenarios. The LSAS-SR has demonstrated good psychometrics in previous research (Baker et al., 2002; dos Santos et al., 2013). In the current study, the LSAS-SR demonstrated strong internal consistency (*α* = 0.95).

The SIAS was also completed again at post-intervention to assess social anxiety.

### Procedure

This procedure was approved by the university Institutional Review Board. Participants were recruited from two university sources: an email listserv and an online volunteer research platform. Prior to study recruitment, we used an online generator to create a random order of study conditions. As participants were consented, they were sequentially assigned to this condition list. Participants completed the study session over Zoom with a trained research assistant and were instructed to complete all measures and tasks on the Qualtrics form. Participants were randomized to one of the three conditions, completed demographic information, SIAS, and FPES (in a fixed order), and then completed the FIBM baseline session, FIBM training session, and FIBM post-test session. Participants were blinded to study condition; research assistants were not to ensure participants completed the correct survey. Afterwards, participants completed the LSAS-SR and then SIAS. Participants were then debriefed on the purpose of the study and compensated with their choice of half a credit in a psychology course or $5. During the debriefing, the research assistant informed the participant of their condition assignment, the purpose of the study, the goal for each condition, gave support resources for anxiety, and provided a citation for a past FIBM research paper (Peters et al., 2017). Trial was registered at https://doi.org/10.17605/OSF.IO/XS7JE on 5/14/25.

## Results

### Descriptive statistics

Data are available at https://doi.org/10.17605/OSF.IO/XS7JE. All variables fell in acceptable range for skewness (<∣2∣) and kurtosis (<∣4∣). Descriptive statistics for questionnaires (FPES, SIAS, and LSAS-SR) can be found in Table 2. Baseline SIAS (*M* = 30.16) suggest the sample had higher levels of social anxiety symptoms than unselected samples (*M* = 19), and similar levels to clinical samples (*M* = 34.6; Mattick and Clarke, 1998). This level of social anxiety is comparable to Rawdon et al. (2018), which observed clinically elevated levels of baseline social anxiety in their sample (*M* ≥ 29 for intervention and control groups) as measured by the SPAI-C.

**Table 2:** Descriptive statistics for baseline and post-intervention measures by condition.

Measures	Positive		Negative		Accurate		Total sample	
Baseline	M	SD	M	SD	M	SD	M	SD
- FIBM^*^	6.88	2.03	6.78	2.13	11.17	1.21	–	–
- SIAS	29.60	15.05	28.73	15.48	31.61	14.74	30.16	14.90
- FPES	32.53	14.15	28.43	11.83	33.61	13.52	31.36	13.20
Post-intervention								
- FIBM^*^	8.30	2.01	5.79	1.57	12.09	1.16	–	–
- SIAS	31.07	17.13	29.35	16.40	33.22	16.51	31.42	16.59
- LSAS-SR	59.20	27.93	57.81	25.63	60.67	26.14	60.15	26.28

* FIBM total sample scores cannot be calculated due to different scoring for the accurate condition compared to the positive and negative condition.

### Group differences

To assess for differences between conditions at baseline, a chi-square test for independence for categorical variables and univariate analyses of variance (ANOVAs) for continuous variables were conducted. There were no differences between conditions in gender [*X*^2^ (8, *N* = 139) = 0.72 *p* = 0.653], ethnicity [*X*^2^ (2, *N* = 139) = 0.27, *p* = 0.277], or race [*X*^2^ (8, *N* = 139) = 0.57, *p* = 0.512]. However, the accurate condition had an average age of 20.62 years old, which was significantly younger than the negative condition (24.13 years old) [*F*(2, 136) = 4.11, *p* = 0.019, *η*_p_^2^ = 0.06], but not significantly different than the positive condition (22.87 years old). Given the significant age difference between the accurate condition group and the negative condition group, all analyses were rerun with age as a covariate and the pattern of significance within results did not change. Therefore, we present analyses without age as a covariate. There were no significant differences between conditions at baseline for FPES [*F*(2, 134) = 1.91, *p* = 0.153, *η*_p_^2^ = 0.03], SIAS [*F*(2, 134) = 0.43 *p* = 0.649, *η*_p_^2^ = 0.01], or FIBM score [*t*(90) = 0.23, *p* = 0.815, Cohen’s *d* = 0.05]; FIBM score could only be compared positive and negative conditions.

### FIBM effects on interpretation of faces

To assess the effect of condition on FIBM scores, a repeated measures ANOVA with condition (positive, negative) as the between-subject variable and phase (baseline, post-test) as the within-subject variable was conducted. There was a main effect of condition [*F*(1, 90) = 13.85, *p* < 0.001, *η*_p_^2^ = 0.13], such that participants in the positive condition had higher FIBM scores than participants in the negative condition. There was not a main effect of phase [*F*(1, 90) = 1.20, *p* = 0.277, *η*_p_^2^ = 0.01].

**Figure 2:**
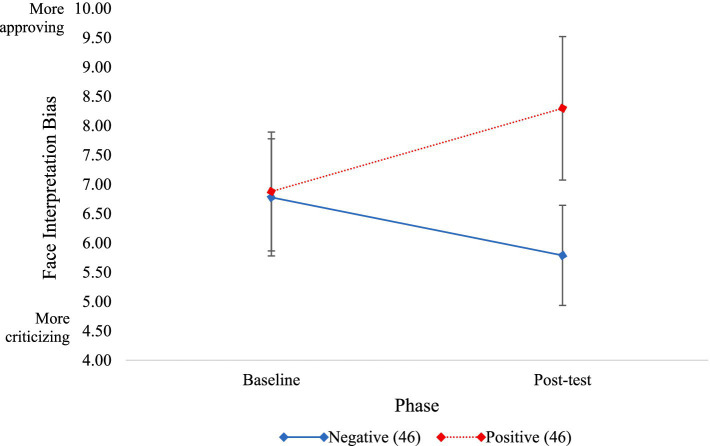
Significant changes in FIBM score from baseline to post-test for negative and positive conditions. Error bars represent standard error of the mean.

In line with hypotheses, there was a significant phase by condition interaction [*F*(1, 90) = 66.96, *p* < 0.001, *η*_p_^2^ = 0.28]. At post-test, participants in the positive condition had significantly greater FIBM scores than participants in the negative condition, *t*(90) = 6.69, *p* < 0.001, Cohen’s *d* = 1.40. Within the positive condition, participants’ FIBM scores significantly increased following FIBM, *t*(46) = 5.13, *p* < 0.001, Cohen’s *d* = 0.71. Within the negative condition, participants’ FIBM scores significantly decreased following FIBM, *t*(46) = 3.36, *p* = 0.002, Cohen’s *d* = 0.53 (see Figure 2).

To assess if the accurate condition led to more accurate interpretations of faces, a paired samples *t*-test was conducted. As hypothesized, participants in the accurate condition were significantly more accurate at identifying facial expressions at post-test compared to baseline, *t*(45) = 4.58, *p* = <0.001, Cohen’s *d* = 0.78.

### FIBM effects on social anxiety

**Figure 3:**
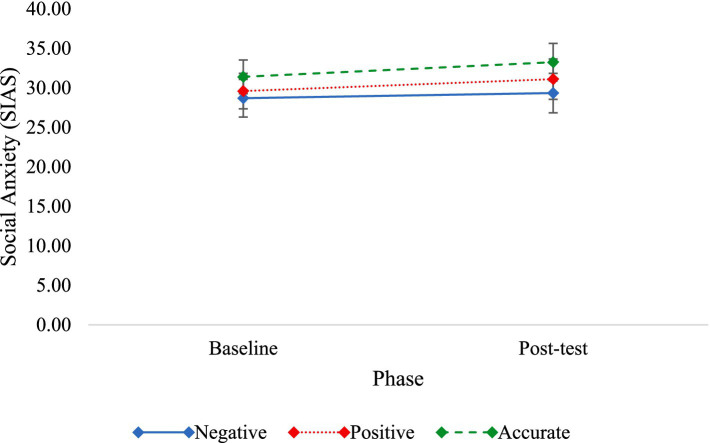
Non-significant changes in social anxiety from baseline to post-test by condition. Error bars represent standard error of the mean.

To assess if FIBM affected SIAS scores from baseline to post-intervention, a repeated measures ANOVA with condition (positive, negative, accurate) as the between-subject variable and phase (baseline, post-test) as the within-subject variable was conducted. Results demonstrated there was a significant effect of phase on SIAS scores in which SIAS scores at post-test were significantly greater than SIAS scores at baseline [*F*(1, 132) = 8.56, *p* = 0.004, *η*_p_^2^ = 0.06]. There was not a main effect of condition [*F*(2, 132) = 0.51, *p* = 0.601, *η*_p_^2^ = 0.01]. Contrary to hypotheses, there was not a significant phase by condition interaction for SIAS scores [*F*(2, 132) = 0.60, *p* = 0.549, *η*_p_^2^ = 0.01] (see Figure 3).

To assess if there were differences between groups for post-intervention LSAS-SR scores, a one-way ANOVA was conducted with condition (positive, negative, accurate) as the between-subject variable. Similar to SIAS scores, there was not a significant difference between groups for Total LSAS-SR scores, [*F*(2, 132) = 0.07, *p* = 0.928, *η*_p_^2^ < 0.01].

### Exploratory moderator analyses

Exploratory analyses were conducted to assess if FPES or baseline SIAS moderated the relationship between FIBM condition and outcome variables. We conducted a series of mixed-model ANOVAs with one between-subjects variable (condition: positive, negative), one within-subjects variable (phase: baseline, post-test), and one continuous moderator (FPES) for each of our outcomes (SIAS, LSAS-SR, FIBM score shift). Results revealed FPES did not moderate condition effects on any analyses (all *p* > 0.141, all *η*_p_^2^ ≤ 0.03). Similarly, we conducted a series of mixed-model ANOVAs with one between-subjects variable (condition: positive, negative), one within-subjects variable (phase: baseline, post-test), and one continuous moderator (baseline SIAS) for each of our outcomes (SIAS, LSAS-SR, FIBM score shift). Results revealed baseline SIAS (all *p* > 0.196; all *η*_p_^2^ ≤ 0.04) did not moderate condition effects on any analyses.

### Correlation analyses

To assess the expected relationships between measures, Pearson correlations were conducted (see Table 3). As expected, baseline FIBM score was significantly correlated to post-test FIBM score and social anxiety measures were inter-correlated. Surprisingly, FIBM scores were not significantly correlated to social anxiety measures.

**Table 3:** Correlation coefficients calculated for baseline and outcome variables.

Variable	FIBM base	FIBM post	FIBM Acc base	FIBM Acc post	FPES	Baseline SIAS	Post SIAS	SIAS change	LSAS anxiety	LSAS avoid	LSAS total
FIBM base	1										
FIBM post	0.43**	1									
FIBM Acc Base	–	–	1								
FIBM Acc post	–	–	0.33*	1							
FPES	−0.10	0.05	−0.06	−0.05	1						
Baseline SIAS	−0.15	−0.15	0.11	−0.12	0.62**	1					
Post SIAS	−0.13	−0.12	0.18	−0.11	0.65**	0.95**	1				
SIAS change	0.06	0.07	0.23	−0.01	0.28**	0.13	0.44**	1			
LSAS anxiety	−0.20	−0.18	0.09	−0.09	0.63**	0.82**	0.85**	0.35**	1		
LSAS avoid	−0.11	−0.18	0.24	−0.01	0.54**	0.75**	0.78**	0.32**	0.83**	1	
LSAS total	−0.16	−0.18	0.17	−0.05	0.61**	0.83**	0.86**	0.35**	0.96**	0.951**	1

FIBM Base = baseline score for face interpretation bias modification task for positive and negative conditions.

FIBM Post = post-test score for face interpretation bias modification task for positive and negative conditions.

FIBM Acc Base = baseline score for face interpretation bias modification task for accurate condition.

FIBM Acc Post = post-test score for face interpretation bias modification task for accurate condition.

**Correlation is significant at the 0.01 level (2-tailed).

*Correlation is significant at the 0.05 level (2-tailed).

## Discussion

The current study replicated and extended previous research by testing the effect of FIBM on interpretations of ambiguous faces and social anxiety symptoms in an unselected college sample. Results revealed FIBM successfully shifted interpretation of ambiguous faces in expected directions for all conditions, but such shifts did not affect social anxiety. In line with hypotheses, FIBM positive condition did lead to more positive interpretations of ambiguous faces. This is in line with similar FIBM studies demonstrating the effectiveness of a positive condition (Penton-Voak et al., 2012; Rawdon et al., 2018; Van Meter et al., 2021). This supports the effectiveness of FIBM in a single-session, online setting, which had not previously been studied in the literature. Additionally, these results suggest the interpretation of ambiguous faces is malleable. Participants’ knowledge of study aims or hypotheses was not assessed, so researchers are unable to rule out demand characteristics.

This is the first study to evaluate negative and accurate conditions within FIBM, and, as hypothesized, both conditions were successfully impacted ambiguous face interpretation. Therefore, FIBM can test if changes in interpretations can lead to both symptom reduction and symptom exacerbation. This finding also opens other avenues for research given negative interpretation biases are implicated in other disorders, like depression (Bourke et al., 2010; Disner et al., 2011).

Contrary to the present hypotheses, shifts in interpretation biases did not lead to shifts in social anxiety. This result was surprising given Rapee and Heimberg (1997) included audience evaluation as one of the core tenets in the social anxiety cognitive model. Though we are hesitant to interpret null results, this suggests the interpretation of ambiguous faces may not be causally related to social anxiety, and thus this cognitive model may need modification. The null finding matches similar null FIBM results on mood (Kuin et al., 2020; Penton-Voak et al., 2020; Peters et al., 2017).

Given Rawdon et al. (2018) found an impact on depressive symptoms at two-week follow up in their study on social anxiety, it’s possible the modified FIBM in this study may have impacted other mood measures (e.g., depression, anger), but such measurements were not collected in the current study. It may be FIBM is better suited to depressive symptoms and therefore to target depression rather than social anxiety. However, other research has attempted to use FIBM to impact depressive symptoms and has not been successful (Penton-Voak et al., 2012).

Additionally, studies that found effects on mood measures also included younger samples (either adolescents and/or young adults) (Penton-Voak et al., 2013; Rawdon et al., 2018; Van Meter et al., 2021). It’s possible FIBM might have a stronger impact on mood measures in younger samples because their mood and psycho/social attributes might be more sensitive to change in cognitive biases. Future research should attempt to investigate this pattern by studying even younger children (e.g., pre-adolescents).

Surprisingly, the current study did not find correlations between FIBM scores and social anxiety measures at baseline. This may indicate the composite faces chosen for this study, or the emotions selected (happy, labeled as “approving,” and angry, labeled as “criticizing”) were not appropriate measures to represent the audience reaction piece in the cognitive model by Rapee and Heimberg (1997). On the other hand, this lack of a relationship may also indicate the interpretation of ambiguous faces is not associated with SAD, and calls into question this tenant of Rapee and Heimberg’s cognitive model (1997).

The null results of FIBM on social anxiety may be due to methodological limitations associated with a single session and/or online design. CBM research has found that multiple sessions and in person interventions (compared to single-session and online format) lead to larger effects (Beard et al., 2012; Fodor et al., 2020; Hakamata et al., 2010; Hallion and Ruscio, 2011). Further research should examine multiple in person sessions of the current modified FIBM. Additionally, a longer delay between FIBM post-test phase and social anxiety measurement may lead to FIBM effects on social anxiety symptoms. Researchers have not yet assessed the psychometric properties of FIBM tasks, and it would be beneficial for future FIBM research to evaluate task reliability and validity. Future mediation studies are also needed to see if symptom change (when it occurs) in FIBM tasks is due to change in facial interpretation bias. To the best of our knowledge, this has not yet been done.

Alternatively, specific methodological choices related to our FIBM task may have led to null findings on social anxiety. For example, in the positive and negative conditions, feedback intended to shift interpretations was only provided for the five faces in the middle of the happy to angry spectrum; feedback to shift interpretations for more faces may strengthen effects. Additionally, the study did not use individualized balance points used in previous studies (Kuin et al., 2020; Penton-Voak et al., 2012; Penton-Voak et al., 2020; Peters et al., 2017; Rawdon et al., 2018; Van Meter et al., 2021). Therefore, it’s possible the feedback was corrective for different numbers of faces for different participants in the same condition. Finally, our questionnaire selection may have affected results: using the same measure (SIAS) twice in the same brief session or using a measure that relies on daily experiences (LSAS-SR) may have reduced our ability to see FIBM effects on social anxiety.

Exploratory analyses revealed that fear of positive evaluation and baseline social anxiety did not moderate FIBM effectiveness at shifting facial interpretations. These findings suggest FIBM can impact interpretations regardless of baseline fear of evaluation and social anxiety.

Results should be interpreted in light of several limitations. First, to be consistent with previous FIBM studies (Kuin et al., 2020; Penton-Voak et al., 2012; Penton-Voak et al., 2020; Peters et al., 2017; Van Meter et al., 2021), the same faces were used for pre-test, training, and post-test. Therefore, we were unable to assess generalization of training effects to novel faces. The use of novel faces for post-test would be a useful future direction for research. Given that we used the same faces at pre-test and post-test and did not include a delay between training and post-test, it’s possible that we may have trained a response bias, rather than shifting face interpretation bias. Second, the faces used were morphed/composite faces (as opposed to real/natural faces), which may also limit generalizability to real world applications. Third, as the current study was conducted online, we are unable to control participant engagement and attention, which may have influenced data quality. Fourth, this study did not measure state affect; it is possible that groups had different baseline levels of state anxiety, which could have affected FIBM results. Fifth, the current study was limited in its post-intervention outcome measures. It would have been interesting to add a related construct of social anxiety, such as the fear of negative evaluation. Finally, the current sample was composed of university students, majority white, and majority female-identifying. The current study population also skewed more female (63%), White (70%), and non-Hispanic (93%) than the general undergraduate population in the United States (57% female, 52% White, 79% non-Hispanic; Hanson, 2025). This demographic composition limits the generalizability of results to other samples.

In conclusion, the present study successfully tested the negative and accurate conditions in FIBM and modified facial interpretations in a single online session. However, FIBM did not impact social anxiety. This challenges the importance of evaluating audience reaction in the Rapee and Heimberg (1997) cognitive model of social anxiety.

## Data Availability

The datasets presented in this study can be found in online repositories. The names of the repository/repositories and accession number(s) can be found at: doi: 10.17605/OSF.IO/XS7JE.

## References

[ref1] American Psychiatric Association (2013). Diagnostic and Statistical Manual of Mental Disorders. Fifth Edn American Psychiatric Association.

[ref2] Baker S. L. Heinrichs N. Kim H.-J. Hofmann S. G. (2002). The Liebowitz social anxiety scale as a self-report instrument: a preliminary psychometric analysis. Behav. Res. Ther. 40, 701–715. doi: 10.1016/S0005-7967(01)00060-2, 12051488

[ref3] Beard C. Sawyer A. T. Hofmann S. G. (2012). Efficacy of attention bias modification using threat and appetitive stimuli: a meta-analytic review. Behav. Ther. 43, 724–740. doi: 10.1016/j.beth.2012.01.002, 23046776 PMC3494088

[ref4] Beck A. Emery G. Greenberg R. (1985). Anxiety disorders and phobias. A cognitive perspective (pp. 300–368). New York: Basic Books.

[ref5] Bourke C. Douglas K. Porter R. (2010). Processing of facial emotion expression in major depression: a review. Aust. N. Z. J. Psychiatry 44, 681–696. doi: 10.3109/00048674.2010.496359, 20636189

[ref6] Carter M. M. Sbrocco T. Tang D. Rekrut F. M. Condit C. (2014). Psychometric properties of the social phobia and social interaction anxiety scales: evidence of construct equivalence in an African American sample. J. Anxiety Disord. 28, 633–643. doi: 10.1016/j.janxdis.2014.07.003, 25124500

[ref7] Cui L. Dong X. Zhang S. (2021). ERP evidence for emotional sensitivity in social anxiety. J. Affect. Disord. 279, 361–367. doi: 10.1016/j.jad.2020.09.111, 33099050

[ref8] Disner S. G. Beevers C. G. Haigh E. A. P. Beck A. T. (2011). Neural mechanisms of the cognitive model of depression. Nat. Rev. Neurosci. 12, 467–477. doi: 10.1038/nrn3027, 21731066

[ref9] dos Santos L. F. Loureiro S. R. Crippa J. A. S. de Lima Osório F. (2013). Adaptation and initial psychometric study of the self-report version of Liebowitz Social Anxiety Scale (LSAS-SR). Int. J. Psychiatry Clin. Pract. 17, 139–143. doi: 10.3109/13651501.2012.710336, 22809130

[ref10] Faul F. Erdfelder E. Lang A.-G. Buchner A. (2007). G*Power 3: a flexible statistical power analysis program for the social, behavioral, and biomedical sciences. Behav. Res. Methods 39, 175–191. doi: 10.3758/BF03193146, 17695343

[ref11] Fodor L. A. Georgescu R. Cuijpers P. Szamoskozi Ş. David D. Furukawa T. A. . (2020). Efficacy of cognitive bias modification interventions in anxiety and depressive disorders: a systematic review and network meta-analysis. Lancet Psychiatry 7, 506–514. doi: 10.1016/S2215-0366(20)30130-9, 32445689

[ref12] Hakamata Y. Lissek S. Bar-Haim Y. Britton J. C. Fox N. A. Leibenluft E. . (2010). Attention bias modification treatment: a meta-analysis toward the establishment of novel treatment for anxiety. Biol. Psychiatry 68, 982–990. doi: 10.1016/j.biopsych.2010.07.021, 20887977 PMC3296778

[ref13] Hallion L. S. Ruscio A. M. (2011). A meta-analysis of the effect of cognitive bias modification on anxiety and depression. Psychol. Bull. 137, 940–958. doi: 10.1037/a0024355, 21728399

[ref14] Hanson M. (2025). College enrollment & student demographic statistics. EducationData.org, 2025-03-17. Available online at: https://educationdata.org/college-enrollment-statistics (Accessed March 7, 2026).

[ref15] Heeren A. Mogoașe C. Philippot P. McNally R. (2015). Attention bias modification for social anxiety: a systematic review and meta-analysis. Clin. Psychol. Rev. 40, 76–90. doi: 10.1016/j.cpr.2015.06.001, 26080314

[ref16] Jones E. B. Sharpe L. (2017). Cognitive bias modification: a review of meta-analyses. J. Affect. Disord. 223, 175–183. doi: 10.1016/j.jad.2017.07.034 28759865

[ref17] Krebs G. Pile V. Grant S. Degli Esposti M. Montgomery P. Lau J. Y. F. (2018). Research review: cognitive bias modification of interpretations in youth and its effect on anxiety: a meta-analysis. J. Child Psychol. Psychiatry 59, 831–844. doi: 10.1111/jcpp.12809, 29052837

[ref18] Kuin N. C. Masthoff E. D. M. Nunnink V. N. Munafò M. R. Penton-Voak I. S. (2020). Changing perception: a randomized controlled trial of emotion recognition training to reduce anger and aggression in violent offenders. Psychol. Violence 10, 400–410. doi: 10.1037/vio0000254

[ref19] Liu H. Li X. Han B. Liu X. (2017). Effects of cognitive bias modification on social anxiety: a meta-analysis. PLoS One 12:e0175107. doi: 10.1371/journal.pone.0175107, 28384301 PMC5383070

[ref20] Lundqvist D. Flykt A. Öhman A. (1998). The Karolinska Directed Emotional Faces—KDEF [CD-ROM]. Stockholm: Department of Clinical Neuroscience, Karolinska Institutet.

[ref21] MacLeod C. Mathews A. (2012). Cognitive bias modification approaches to anxiety. Annu. Rev. Clin. Psychol. 8, 189–217. doi: 10.1146/annurev-clinpsy-032511-143052, 22035241

[ref22] Mattick R. P. Clarke J. C. (1998). Development and validation of measures of social phobia scrutiny fear and social interaction anxiety. Behav. Res. Ther. 16, 455–470. 10.1016/s0005-7967(97)10031-6 9670605

[ref23] Penton-Voak I. S. Adams S. Button K. S. Fluharty M. Dalili M. Browning M. . (2020). Emotional recognition training modifies neural response to emotional faces but does not improve mood in healthy volunteers with high levels of depressive symptoms. Psychol. Med. 51, 1211–1219. doi: 10.1017/S0033291719004124, 32063231

[ref24] Penton-Voak I. S. Bate H. Lewis G. Munafò M. R. (2012). Effects of emotion perception training on mood in undergraduate students: randomised controlled trial. Br. J. Psychiatry 201, 71–72. doi: 10.1192/bjp.bp.111.107086, 22539781

[ref25] Penton-Voak I. S. Thomas J. Gage S. H. McMurran M. McDonald S. Munafò M. R. (2013). Increasing recognition of happiness in ambiguous facial expressions reduces anger and aggressive behavior. Psychol. Sci. 24, 688–697. doi: 10.1177/0956797612459657, 23531485

[ref26] Peters S. E. Lumsden J. Peh O. H. Penton-Voak I. S. Munafò M. R. Robinson O. J. (2017). Cognitive bias modification for facial interpretation: a randomized controlled trial of transfer to self-report and cognitive measures in a healthy sample. R. Soc. Open Sci. 4:170681. doi: 10.1098/rsos.170681, 29308221 PMC5749989

[ref27] Possis E. A. Kemp J. J. Lickel J. J. Sy J. T. Dixon L. J. Deacon B. J. (2013). A comparison of cognitive and behavioral approaches for reducing cost bias in social anxiety. J. Cogn. Psychother. 27, 210–220. doi: 10.1891/0889-8391.27.3.210, 32759142

[ref28] Rapee R. M. Heimberg R. G. (1997). A cognitive-behavioral model of anxiety in social phobia. Behav. Res. Ther. 35, 741–756. doi: 10.1016/S0005-7967(97)00022-3, 9256517

[ref29] Rawdon C. Murphy D. Motyer G. Munafò M. R. Penton-Voak I. Fitzgerald A. (2018). An investigation of emotion recognition training to reduce symptoms of social anxiety in adolescence. Psychiatry Res. 263, 257–267. doi: 10.1016/j.psychres.2018.02.023, 29602534

[ref30] Van Meter A. Stoddard J. Penton-Voak I. Munafò M. R. (2021). Interpretation bias training for bipolar disorder: a randomized controlled trial. J. Affect. Disord. 282, 876–884. doi: 10.1016/j.jad.2020.12.162, 33601731 PMC12702476

[ref31] Weeks J. W. Heimberg R. G. Rodebaugh T. L. (2008). The fear of positive evaluation scale: assessing a proposed cognitive component of social anxiety. J. Anxiety Disord. 22, 44–55. doi: 10.1016/j.janxdis.2007.08.002, 17884328

[ref32] Yoon K. L. Zinbarg R. E. (2008). Interpreting neutral faces as threatening is a default mode for socially anxious individuals. J. Abnorm. Psychol. 117, 680–685. doi: 10.1037/0021-843X.117.3.680, 18729619

